# The gray area of RQ-PCR-based measurable residual disease: subdividing the “positive, below quantitative range” category

**DOI:** 10.1038/s41375-024-02265-z

**Published:** 2024-05-17

**Authors:** Michaela Kotrova, Eva Fronkova, Michael Svaton, Daniela Drandi, Felix Schön, Patricia Hoogeveen, Jeremy Hancock, Aneta Skotnicova, Anke Schilhabel, Cornelia Eckert, Emmanuelle Clappier, Gianni Cazzaniga, Beat W. Schäfer, Jacques J. M. van Dongen, Matthias Ritgen, Christiane Pott, Vincent H. J. van der Velden, Jan Trka, Monika Brüggemann

**Affiliations:** 1 Department of Internal Medicine II, University Hospital Schleswig-Holstein, Kiel, Germany; 2 CLIP—Childhood Leukaemia Investigation Prague, Department of Paediatric Haematology and Oncology, Second Faculty of Medicine, Charles University and University Hospital Motol, Prague, Czech Republic; 3 St. Anna Children’s Cancer Research Institute (CCRI), Vienna, Austria; 4 Austrian Academy of Sciences, CeMM Research Center for Molecular Medicine, Vienna, Austria; 5 Hematology Division, Department of Molecular Biotechnology and Health Sciences, University of Torino, Torino, Italy; 6 Department of Immunology, Laboratory Medical Immunology, Erasmus MC, University Medical Center Rotterdam, Rotterdam, Netherlands; 7 Bristol Genetics Laboratory, Southmead Hospital, Bristol, UK; 8 Department of Pediatric Hematology and Oncology, Charité-Universitätsmedizin Berlin, Berlin, Germany; 9 German Cancer Consortium (DKTK), and German Cancer Research Center (DKFZ), Heidelberg, Germany; 10 Hematology Laboratory, Saint Louis Hospital, Assistance Publique-Hôpitaux de Paris (AP-HP), Paris, France; 11 Tettamanti Cente, Fondazione IRCCS San Gerardo dei Tintori, Monza, Italy; 12 Department of Hematology, University Hospital, Zürich, Switzerland; 13 Department of Immunology, Leiden University Medical Center (LUMC), Leiden, the Netherlands

**Keywords:** Genetics research, Translational research

## To the Editor:

Detection of measurable residual disease (MRD) in hematological malignancies is crucial for prognostication and treatment decisions. Real-time quantitative PCR (RQ-PCR), which targets immunoglobulin (IG) and T-cell receptor (TR) rearrangements, is deemed the gold-standard method for MRD detection in national and international clinical trials. However, this approach does have some limitations, primarily related to standard curve performance and non-specific background amplification.

International guidelines and criteria for interpreting IG/TR RQ-PCR-based MRD data, developed by the EuroMRD Consortium and available since 2007 [1], define two key parameters: sensitivity and quantitative range (QR). To ensure reliable and accurate assessment of MRD, sensitivity and QR are determined for each clone-specific RQ-PCR assay using a standard dilution curve of the patient diagnostic sample in leukocytes from healthy donors (so-called “buffy coat” (BC), mimicking the background of polyclonal lymphoid cells in bone marrow samples from patients in complete remission. To minimize the risk of false-positive results, the definition of the sensitivity and QR, as well as the criteria for sample positivity, consider the possible nonspecific background amplification. Additional and more stringent criteria, including reproducibility within replicates and distance from the background, are required to establish the QR and accurately quantify the sample. However, according to these criteria, certain samples may be considered positive, but not quantifiable, also known as “positive below QR” (PBQR).

This category of samples poses a challenge for the interpretation of MRD and subsequent clinical evaluation, potentially leading to an incorrect treatment decision. Previous studies have shown that certain PBQR samples may be false positives, caused by non-specific amplification of IG/TR rearrangements from the patient’s normal lymphocytes [2–4]. On the other hand, samples with true MRD positivity may also fall into this PBQR category due to inconsistent amplification between replicates or poor performance of the patient’s clone-specific standard curve. Therefore, it appears to be necessary to tighten the interpretation guidelines for PBQR results and/or to complement RQ-PCR with other methods that may overcome these limitations.

In this study, we selected PBQR samples from five EuroMRD laboratories (Kiel, Prague, Rotterdam, Torino, and Bristol), for which additional information such as next-generation sequencing (NGS)-MRD, MRD kinetics, or MRD level detected using a second IG/TR marker were available (see Supplementary Table 1 for more details). Please note that the sample selection was strongly influenced by the availability of additional MRD data (NGS, second marker or MRD kinetics) and therefore the cohort composition does not reflect the actual number of PBQR samples for individual diagnostic entities. Our goal was to objectively examine the characteristics of the raw RQ-PCR data of PBQR samples to define new criteria for identifying true MRD positive samples, and to propose an update of the RQ-PCR MRD interpretation guidelines. While the original guidelines solely addressed acute lymphoblastic leukemia (ALL), we have now decided to include also CLL and MCL samples in order to extend the applicability of the guidelines to these entities. A total of 1262 PBQR follow-up samples from patients with ALL, chronic lymphocytic leukemia (CLL) or mantle cell lymphoma (MCL) from the early treatment phases as well as from the later ones and even after transplantation were included in this study (for further details, see Supplementary Table 1). Samples with PBQR RQ-PCR MRD but with a negative benchmark result (either negative NGS-MRD result, negative second IG/TR marker and/or negative MRD kinetics (284/1262, 22.5%)) were assigned to a “probably MRD negative” (pNEG) group. Samples with a positive NGS-MRD result and/or a second IG/TR marker positivity (978/1262, 77.5%) were assigned to a “probably MRD positive” (pPOS) group.

We examined whether additional criteria for stricter evaluation of IG/TR RQ-PCR MRD results could discriminate the pPOS samples from the pNEG samples. These were: i) the difference between the lowest CT of the sample and the background (∆BC), ii) the number of positive sample replicates, and iii) the ΔCT of the sample’s lowest and highest replicate. Table 1 outlines the criteria and summarizes their combinations that we applied in each of the scenarios to the RQ-PCR results.

**Table 1:**
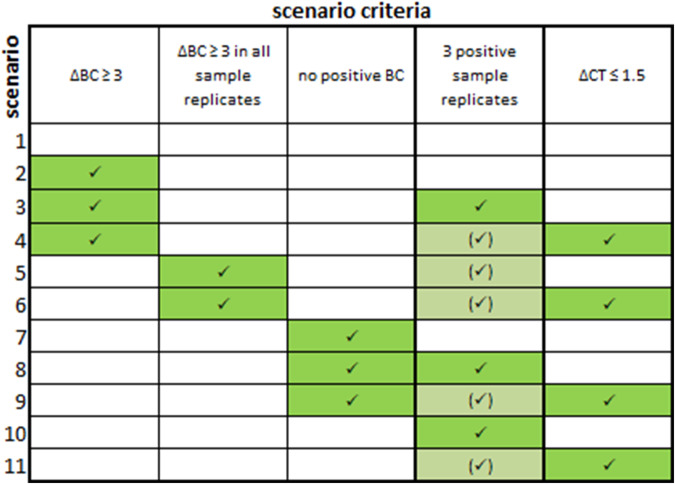
Overview of criteria and their combinations (scenarios) which were applied to the RQ-PCR data.

The table displays the 11 scenarios that we evaluated. For all samples that met the conditions of scenarios requiring either ΔCT < 1.5 or ΔBC ≥ 3 in all sample replicates (scenarios 4, 5, 6, 9, and 10), all three sample replicates were positive (indicated by bracketed ticks). ΔBC, the difference between the sample’s lowest CT and the buffy coat’s (BC) lowest CT; positive sample replicates, the number of positive sample replicates with CT ≥ 1 lower the lowest CT of the BC; ΔCT, CT difference of the sample’s lowest and highest replicate.

For each of the 11 scenarios that we evaluated, we provide information on the number of samples that met its criteria (samples covered) and the proportions of samples that were classified according to these criteria and the results of the benchmark test into the three following groups: 1) true positives (pPOS samples meeting the applied criteria), 2) true negatives (pNEG samples not meeting the criteria), and 3) missed positives (pPOS samples not meeting the criteria). All of the results are comprehensively presented in Supplementary Table 2.

**Fig. 1:**
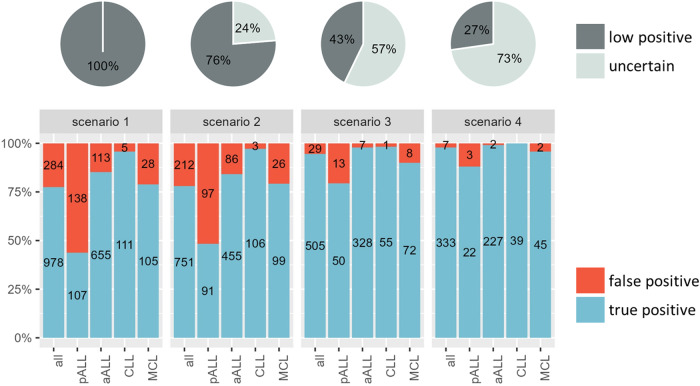
Distribution of samples based on scenario criteria (scenarios 1–4). The upper panel utilizes pie charts to illustrate the proportion of samples satisfying the scenario criteria (assigned as “MRD low positive” shown in dark gray) and those failing to meet the criteria (categorized as “uncertain MRD” shown in light gray). The lower panel employs bar plots to visualize the composition of the “MRD low positive” group. Red bars represent false positive samples (negative benchmark result), while blue bars depict true positive samples (positive benchmark result). Scenario 1 is the baseline scenario showing all PBQR samples without any additional criteria. Scenario 2 including samples with ΔBC ≥ 3 in at least one of the sample replicates is the scenario with highest sensitivity. Scenario 3 with the following criteria: 3 positive sample replicates and ΔBC ≥ 3 in at least one of the sample replicates is the scenario offering the most favorable balance between sensitivity and specificity. Scenario 4 including samples with the ΔCT between sample replicates ≤ 1.5 and ΔBC ≥ 3 in at least one of the sample replicates (all these samples also have 3 positive sample replicates) is the scenario with the highest specificity. The remaining scenarios are depicted in the Supplementary Fig. 1. Abbreviations: all all samples, ALL acute lymphoblastic leukemia (further subdivided into pALL—pediatric and aALL—adult), CLL chronic lymphoblastic leukemia, MCL mantle cell lymphoma.

**Scenario 1** (Fig. 1) was the baseline scenario that applied the original criteria [1] currently used in treatment protocols. This scenario therefore covered all PBQR samples from our cohort; however, it demonstrated low accuracy in identifying truly MRD positive samples, as indicated by the high proportion of pNEG samples (284/1262, 22.5% pNEG and 978/1262, 77.5% pPOS samples). In these pNEG samples, NGS, MRD kinetics, or second IG/TR marker MRD pointed towards MRD negativity of the sample. Our goal was to select among **scenarios 2-11** the one that formed the best compromise of identifying true positives and true negatives, while excluding as few outcomes as possible. We decided on **scenario 3** which required that a sample had three positive replicates with a ΔCT ≥ 1 from the lowest background CT, and the lowest sample replicate CT was ≥ 3 CT lower than the lowest background CT. This scenario resulted in an increase in the true positivity rate from 77.5% (978/1262) to 94.6% (505/534) and covered 42.8% (534/1248) of the PBQR samples (Fig. 1). **Scenario 4** achieved an outstanding true positivity rate of 97.9% (333/340, Fig. 1) applying the same criteria for the distance from the background as in scenario 3, while introducing more stringent criteria for the CT value differences between the sample replicates (ΔCT < 1.5 between the lowest and the highest values). However, this strict scenario covered only about a quarter of the samples (340/1248, 27.2%), therefore was not selected. Note the remarkable similarity of scenario 3 and **scenario 10** (Supplementary Fig. 1), with the main difference being the inclusion of the “∆BC ≥ 3 criterion” in scenario 3. This criterion significantly increases the specificity of classifying results as true positives within potential polyclonal background signals, thus justifying the selection of scenario 3.

Our findings illustrate that implementing more stringent criteria for the interpretation of the RQ-PCR MRD data can enhance the certainty of true MRD positivity. It is important to mention that while pALL samples showed a positive benchmark result in only 44% (107/245) of cases, other entities like aALL (85%, 655/768), CLL (96%, 111/116), and MCL (79%, 106/133) had much higher positivity rates. This variation reflects the underlying disease biology and resulting in different pre-test probability of MRD positivity. While some might argue for entity-specific criteria (e.g., evaluating all CLL PBQR samples as positive due to low false-negative rates), we believe maintaining consistent criteria across entities is crucial to ensure data comparability in future studies and minimize potential interpretation errors.

Furthermore, it was suggested that MRD quantification (as mean of triplicates values) may still be sufficiently accurate in samples fulfilling the scenario 3 criteria. To further investigate this, we examined a cohort of 278 samples (188 aALL for which the information on the 1st and 2nd MRD markers were stored in the Laboratory Information Management System of the Kiel reference laboratory, 5 aALL and 6 pALL with available ddPCR MRD result which were published in an earlier study [5], 26 CLL and 53 MCL samples with available ddPCR MRD data from the Kiel laboratory; 30 of the MCL samples were also included in the main cohort) with three positive sample replicates (positive, below QR) and an available positive (quantifiable) second IG/TR marker or ddPCR-MRD measurements (Supplementary Fig. 2). The difference between these two MRD values was <0.5 log in 210/278 samples (76%), <1 log in 257/278 samples (92%) and >1 log in 21/278 samples (8%), indicating that the calculated RQ-MRD result provides a fair estimate of the MRD level in such samples. Therefore, it was decided that it is allowed to add this estimated MRD level to the report, in between brackets after the “positive, below 10^−x^” (e.g., positive, <10^−4^ (7 × 10^−5^)).

Based on the data provided above, which incorporate NGS-MRD, MRD kinetics and second marker MRD information to corroborate RQ-PCR MRD analysis, and the experience gained by the EuroMRD group over the years of the routine use of IG/TR RQ-PCR for MRD detection, we propose an update of the EuroMRD guidelines for the interpretation of PBQR RQ-PCR MRD results. To enhance the precision of identifying MRD-positive cases and, consequently, to optimize treatment choices and outcomes in patients with hematologic malignancies, the previous category of PBQR samples is now divided into two groups. The first group includes samples that are highly likely positive (meeting the criteria for scenario 3), and these are referred to as “MRD low positive, <QR” (e.g., MRD low positive, <10^−4^). The second group, called ‘MRD of uncertain significance’, includes samples that do not meet the criteria of Scenario 3 and that cannot be reliably classified as truly positive or truly negative. Interestingly, our newly defined criteria for MRD positivity partially overlap with the original definition of positivity on protocols aiming at therapy intensification, which also require at least one of the replicates to be ≥ 3.0 CT lower than the lowest CT of background. Applying other techniques may further support the appropriate classification of such MRD results. NGS-MRD detection has demonstrated both feasibility and accuracy [3, 6–9], and can therefore be used to confirm MRD positivity in PBQR samples. Fragment length analysis is recommended to confirm the appropriate size of the PCR product of PBQR samples whenever possible. If the fragment length is not of the expected size, the sample should be classified as MRD negative. The revised guidelines are published in a separate manuscript that accompanies this paper [10].

### Supplementary information

Supplementary Tables and Figures
