# Supplementary material for: The gray area of RQ-PCR-based measurable residual disease: subdividing the “positive, below quantitative range” category
Source: Leukemia. 2024 May 17;38(7):1617–20. doi: 10.1038/s41375-024-02265-z (PMC11216996; doi:10.1038/s41375-024-02265-z)
Supplement: Supplementary file 1 — Supplementary Tables and Figures [file 41375_2024_2265_MOESM1_ESM.docx]

## Supplementary Information

**The Gray Area of RQ-PCR-based Measurable Residual Disease: Subdividing the 'Positive, Below Quantitative Range' Category**

Michaela Kotrova^1^, Eva Fronkova^2^, Michael Svaton^2,3,4^, Daniela Drandi^5^, Felix Schön^1^, Patricia Hoogeveen^6^, Jeremy Hancock^7^, Aneta Skotnicova^2^, Anke Schilhabel^1^, Cornelia Eckert^8,9^, Emmanuelle Clappier^10^, Gianni Cazzaniga^11^, Beat W Schäfer^12^, Jacques J M van Dongen^13^, Matthias Ritgen^1^, Christiane Pott^1^, Vincent H.J. van der Velden^6^, Jan Trka^2^, Monika Brüggemann^1^

^1^ Department of Hematology, University Hospital Schleswig-Holstein, Kiel, Germany

^2^ CLIP - Childhood Leukaemia Investigation Prague, Department of Paediatric Haematology and Oncology, Second Faculty of Medicine, Charles University and University Hospital Motol, Prague, Czech Republic

^3^ St. Anna Children’s Cancer Research Institute (CCRI), Vienna, Austria

^4^ Austrian Academy of Sciences, CeMM Research Center for Molecular Medicine, Vienna, Austria

^5^ Department of Molecular Biotechnology and Health Sciences, Hematology Division, University of Torino, Torino, Italy

^6^ Laboratory Medical Immunology, Department of Immunology, Erasmus MC, University Medical Center Rotterdam, Rotterdam, Netherlands

^7^ Bristol Genetics Laboratory, Southmead Hospital, Bristol, UK

^8^ Department of Pediatric Hematology and Oncology, Charité-Universitätsmedizin Berlin, Berlin, Germany

^9^ German Cancer Consortium (DKTK), and German Cancer Research Center (DKFZ), Heidelberg, Germany

^10^ Hematology Laboratory, Saint Louis Hospital, Assistance Publique-Hôpitaux de Paris (AP-HP), Paris, France

^11^ Tettamanti Cente, Fondazione IRCCS San Gerardo dei Tintori, Monza, Italy

^12^ Department of Hematology, University Hospital, Zürich, Switzerland

^13^ Department of Immunology, Leiden University Medical Center (LUMC), Leiden, Netherlands.

Supplementary Table 1

|  | pNEG | | | pPOS | |  | | |
| --- | --- | --- | --- | --- | --- | --- | --- | --- |
|  | NGSneg | 2ndMneg | negKIN | NGSpos | 2ndMpos | TOTAL pNEG | TOTAL pPOS | **TOTAL** |
| pediatric ALL | 97 | 41 | 0 | 94 | 13 | 138 | 107 | **245** |
| adult ALL | 0 | 0 | 113 | 0 | 655 | 113 | 655 | **768** |
| CLL | 5 | 0 | 0 | 111 | 0 | 5 | 111 | **116** |
| MCL | 28 | 0 | 0 | 105 | 0 | 28 | 105 | **133** |
| **TOTAL** | **130** | **41** | **113** | **310** | **668** | **284** | **978** | **1262** |

**Supplementary Table 1: Overview of samples included in the study and available benchmark results.** The table includes information on diagnostic entities (ALL, acute lymphoblastic leukemia; CLL, chronic lymphocytic leukemia; MCL, mantle cell lymphoma) and the available benchmark result (pNEG, probably MRD negative; pPOS, probably MRD positive; NGSneg, NGS MRD negative; 2ndMneg, 2^nd^ MRD marker negative; negKIN, negative MRD kinetics (IG/TR target negativity of preceding and following sample, both less than three months before/after PBQR); NGSpos, NGS MRD positive; 2ndMpos, 2^nd^ MRD marker positive).


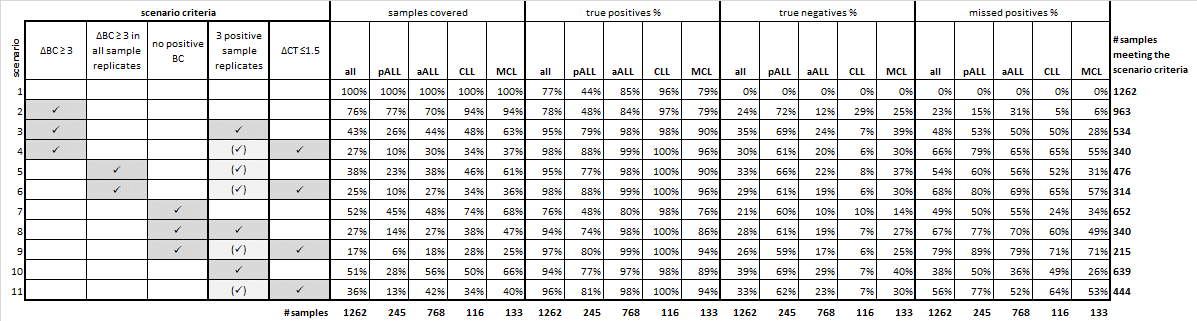
Supplementary Table 2

**Supplementary Table 2: Overview of criteria and their combinations (scenarios) which were applied to the RQ-PCR data, coverage and accuracy compared to benchmark results.** The table displays the 11 scenarios that we evaluated and provides the following values for each scenario across all samples and by diagnostic entity:

“samples covered” [samples fulfilling scenario criteria / all samples; (TP+FP)/(TP+FP+TN+FN)]: Proportion of samples meeting the scenario criteria. This value represents the percentage of samples evaluated under each scenario.

“true positives %” [true positive samples / samples fulfilling the criteria; TP/(TP+FP)]: Proportion of true positive samples within the group meeting the scenario criteria. This metric reflects the accuracy of the scenario in identifying positive samples among those that fulfilled the criteria.

“true negatives %” [true negative samples / samples not fulfilling the criteria; TN/(TN+FN)]: Proportion of true negative samples within the group not meeting the scenario criteria.

“missed positives %” [false positive samples / all pPOS samples; FP/(FP+TP)]: Proportion of positive samples not meeting the scenario criteria within the group of samples with a positive benchmark result.

The percentage of samples with a positive benchmark result (TP samples in Scenario 1) varies significantly across diagnostic entities. This variation considerably impacts the true positive rate observed in other scenarios. For all samples that met the conditions of scenarios requiring either ΔCT < 1.5 or ΔBC ≥ 3 in all sample replicates (scenarios 4, 5, 6, 9, and 10), all three sample replicates were positive (indicated by bracketed ticks). ΔBC, the difference between the sample’s lowest CT and the buffy coat’s (BC) lowest CT; positive sample replicates, the number of positive sample replicates with CT ≥1 lower the lowest CT of the BC; ΔCT, CT difference of the sample’s lowest and highest replicate; pALL, pediatric acute lymphoblastic leukemia; aALL, adult acute lymphoblastic leukemia; CLL, chronic lymphocytic leukemia; MCL, mantle cell lymphoma; TP, true positive samples; FP, false positive samples; TN, true negative samples; FN, false negative samples.


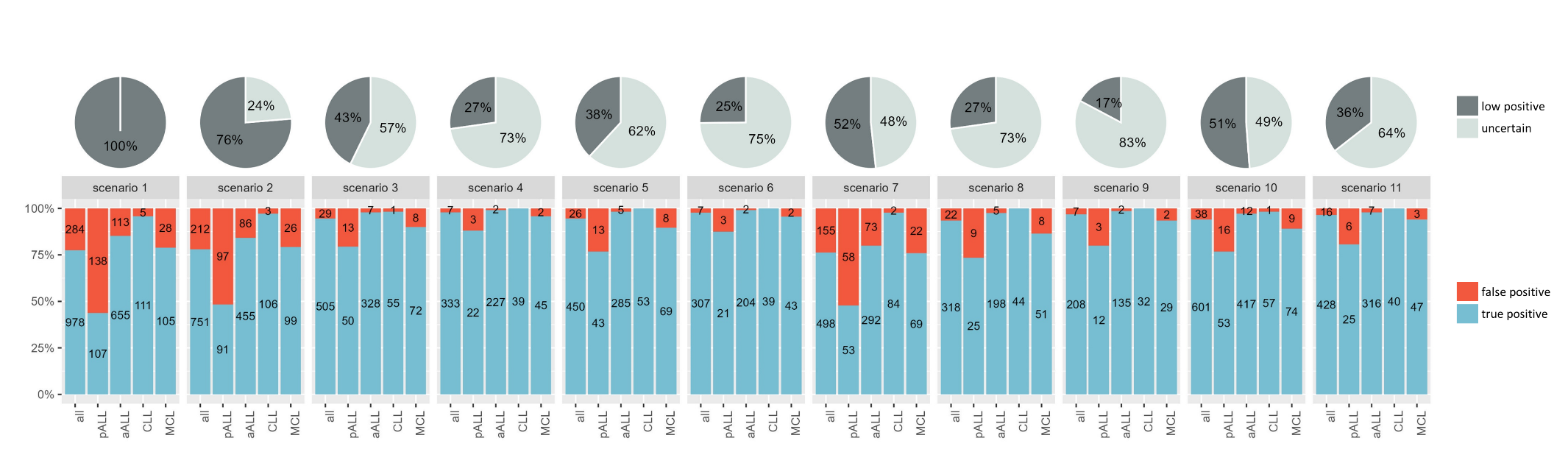
Supplementary Figure 1

**Supplementary Figure 1: Distribution of Samples Based on Scenario Criteria.** This figure depicts the distribution of samples according to whether they meet the criteria outlined in Scenarios 1-11 (detailed in Table 1). The upper panel utilizes pie charts to illustrate the proportion of samples satisfying the scenario criteria (assigned as "MRD low positive," shown in dark grey) and those failing to meet the criteria (categorized as "uncertain MRD," shown in light grey). The lower panel employs bar plots to visualize the composition of the "MRD low positive" group. Red bars represent false positive samples (negative benchmark result), while blue bars depict true positive samples (positive benchmark result). Abbreviations: all, all samples; ALL, acute lymphoblastic leukemia (further subdivided into pALL - pediatric and aALL - adult); CLL, chronic lymphoblastic leukemia; MCL, mantle cell lymphoma.

Supplementary Figure 2

1.
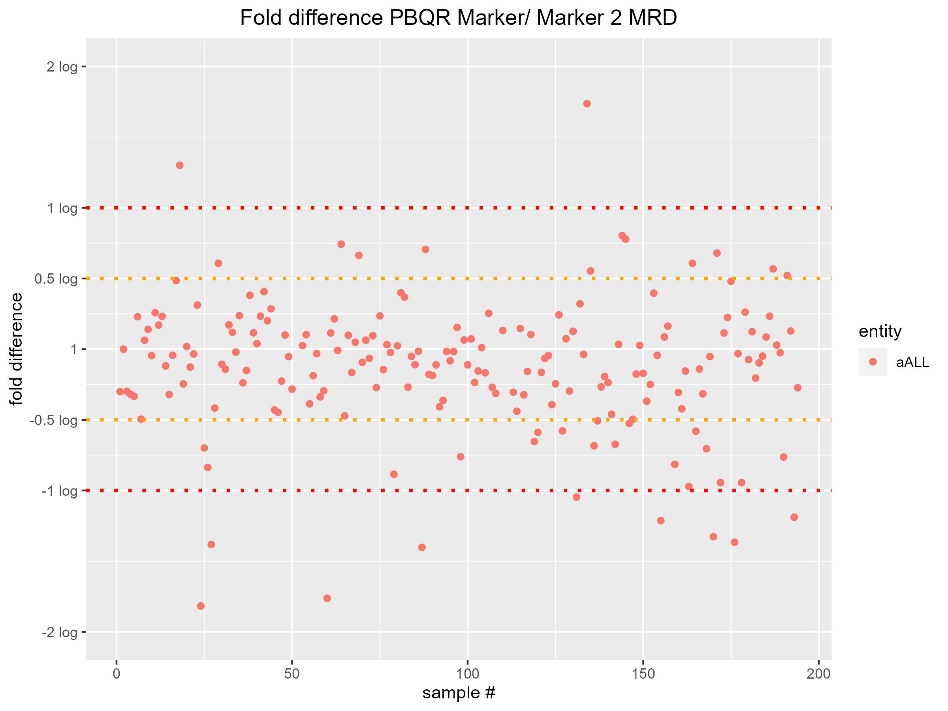

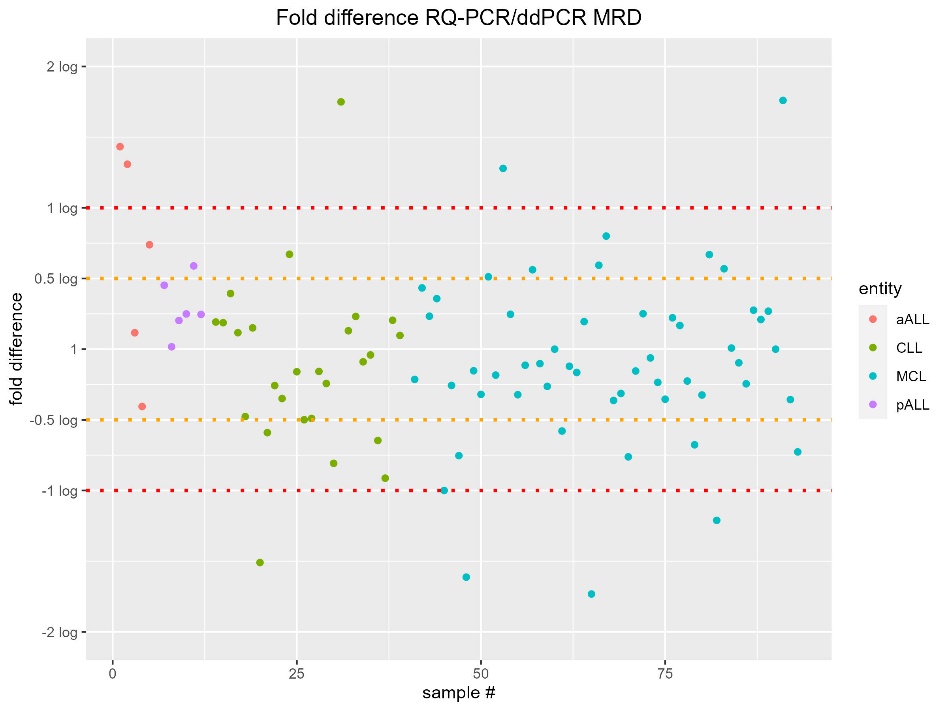
B)

**Supplementary Figure 2: Comparison of PBQR RQ-PCR-based MRD values and values obtained by ddPCR or a second RQ-PCR quantifiable IG/TR MRD marker.** Only PBQR samples with three positive sample replicates in RQ-PCR included. A) Fold difference between RQ-PCR and ddPCR in 90 samples on a log scale. In total, 128 samples with available ddPCR data and with three positive sample replicates despite being PBQR by RQ-PCR were collected (10 aALL, 6 pALL, 74 MCL, 38 CLL). Samples with a negative or non-quantifiable ddPCR MRD result (n=38, 5 aALL, 21 MCL, 12 CLL) were excluded from the analysis. B) Fold difference between the MRD value of a PBQR RQ-PCR marker and a second quantifiable RQ-PCR marker in adult ALL samples (n=188). Six samples with clonal evolution (identified by detailed analysis of the MRD kinetics of both markers) were excluded from the original cohort (n=194). Half-log - orange dotted lines, one-log - red dotted lines. RQ-PCR, real-time quantitative PCR; ddPCR, digital droplet PCR; aALL, adult acute lymphoblastic leukemia, pALL, pediatric acute lymphoblastic leukemia; CLL, chronic lymphocytic leukemia; MCL, mantle cell lymphoma.
